# Clinical application of the Panbio™ COVID-19 Ag rapid test device and SSf-COVID19 kit for the detection of SARS-CoV-2 infection

**DOI:** 10.1186/s13104-022-06226-6

**Published:** 2022-12-05

**Authors:** Sang-Min Oh, Jee-Soo Lee, Hyeon Jae Jo, Donghwan Kim, Dohyeon Park, Young Hoon Hwang, Yunsang Choi, Chan Mi Lee, Seungjae Lee, Euijin Chang, Eunyoung Lee, Taek Soo Kim, Moon-Woo Seong, Pyoeng Gyun Choe, Nam Joong Kim

**Affiliations:** 1 Department of Internal Medicine, Seoul National University College of Medicine, 101 Daehakro, Jongno-gu, 03080 Seoul, Republic of Korea; 2 Department of Laboratory Medicine, Seoul National University College of Medicine, 101 Daehakro, Jongno-gu, 03080 Seoul, Republic of Korea; 3 Division of Infectious Diseases, Seoul Metropolitan Government – Seoul National University Boramae Medical Center, 20 Boramae-ro 5-gil, Dongjak-gu, 07061 Seoul, Republic of Korea; 4 Present Address: Department of Internal Medicine, Jeonbuk National University Medical School and Hospital, 20 Geonjiro, Deokjin-gu, 54907 Jeonju, Jeollabuk-do Republic of Korea

**Keywords:** COVID-19, SARS-CoV-2, RT-PCR, Saliva, Antigen

## Abstract

**Objective:**

We evaluated the sensitivity and specificity of the Panbio™ COVID-19 Ag rapid test device using nasal swabs and those of the SSf-COVID19 kit, one of RT-PCR tests, using saliva specimens. These tests were compared with RT-PCR tests using nasopharyngeal swabs for the diagnosis of SARS-CoV-2 infection. The three diagnostic tests were simultaneously conducted for patients aged ≥ 18 years, who were about to be hospitalized or had been admitted for COVID-19 confirmed by RT-PCR in two research hospitals from August 20 to October 29, 2021. Nasal swabs were tested using the Panbio™ COVID-19 Ag rapid test device. More than 1 mL of saliva was self-collected and tested using the SSf-COVID19 kit.

**Results:**

In total, 157 patients were investigated; 124 patients who were about to be hospitalized and 33 patients already admitted for COVID-19. The overall sensitivity and specificity of the Panbio™ COVID-19 Ag rapid test device with nasal swabs were 64.7% (95% confidence interval [CI] 47.9–78.5%) and 100.0% (95% CI 97.0–100.0%), respectively. The median time to confirm a positive result was 180 s (interquartile range 60–255 s). The overall sensitivity and specificity of the SSf-COVID19 kit with saliva specimens were 94.1% (95% CI 80.9–98.4%) and 100.0% (95% CI 97.0–100.0%), respectively.

## Introduction

Reverse transcription-polymerase chain reaction (RT-PCR) assay of nucleic acids using upper and/or lower respiratory specimens is the most widely used method for diagnosing severe acute respiratory syndrome coronavirus 2 (SARS-CoV-2) infection [1, 2]. Despite its high sensitivity and specificity, RT-PCR has disadvantages such as high cost, long turn-around time, and the need for equipment for testing [2, 3]. Rapid antigen tests (RATs) that detect SARS-CoV-2 antigens can be used for point-of-care diagnosis of coronavirus disease (COVID-19) [4–7]. Although RATs have the advantages of low cost, short turn-around time, and lack of instrument requirements for testing, their sensitivity in detecting SARS-CoV-2 is lower than that of RT-PCR, especially for clinical specimens with low viral loads [5–7]. Various nucleic acid amplification tests (NAAT), including RT-PCR or rapid isothermal amplification such as loop-mediated isothermal amplification are now recommended as diagnostic methods for the detection of SARS-CoV-2 [7, 8].

A nasopharyngeal (NP) swab is the standard sample collected for diagnosis because of its high sensitivity and specificity. However, it is relatively invasive, time consuming, and requires trained healthcare workers for sample collection, which are limitations for conducting repeated examinations [2, 3]. Although NP specimens remain the recommended samples for SARS-CoV-2 diagnostic testing, nasal (anterior nares or mid-turbinate) swabs or saliva specimens are acceptable alternatives [9].

In the era of the COVID-19 pandemic, school classes, religious activities, and other activities involving gatherings of people have been partly or fully restricted in the Republic of Korea [10]. As the current pandemic situation is prolonged, various measures are being taken to re-open schools worldwide. To prevent outbreaks in schools, repeated screening tests using rapid diagnostics and non-invasive samples like saliva specimens are considered as a method to support their safe re-opening [11]. This study aimed to evaluate the performance of the Panbio™ COVID-19 Ag rapid test device, a RAT that uses nasal swab specimens, and the SSf-COVID19 kit, a RT-PCR that uses self-collected saliva specimens, in comparison with RT-PCR tests that use NP swabs in the detection of SARS-CoV-2.

## Methods

### Study design

The performance of the Panbio™ COVID-19 Ag rapid test device (Abbott Diagnostic GmbH, Jena, Germany) using nasal swabs and the SSf-COVID19 kit (SEASUN BIOMATERIALS, Inc. Daejeon, Korea) using saliva specimens were compared with the RT-PCR test using NP swabs from August 20 to October 29, 2021. We conducted the three diagnostic tests simultaneously for patients aged ≥ 18 years who were about to be hospitalized at Seoul National University Hospital (SNUH) or were already admitted to SNUH or Seoul Metropolitan Government – Seoul National University Boramae Medical Center (SMG-SNUBMC) for COVID-19 confirmed by RT-PCR.

### Sample collection and diagnostic tests

NP swabs were collected by a skilled nurse or a doctor. RT-PCR with NP swabs was performed using the Standard M nCoV Real-Time Detection kit (SD Biosensor, Inc. Suwon, Korea). Nasal swabs were collected by a skilled doctor, and the specimens were tested using the Panbio™ COVID-19 Ag rapid test device. For the sample collection from the nasal cavities, it is recommended that the swab be inserted less than 1 inch in the nostril, turbinated five times, and then inserted in the test tubes [12]. The time required to interpret the RAT was measured, and the results were observed after 60 min to determine whether the initial results changed during the extended period. More than 1 mL of saliva was collected in a sterile bottle. Before collecting the saliva specimen, a doctor educated the patients about collecting methods, such as open mouth and drooling, to avoid stimulation of the salivary gland. RT-PCR with saliva specimens was performed using SSf-COVID19 kit.

### Statistical analysis

The sensitivity and specificity of the RAT using nasal specimens and the RT-PCR using saliva specimens were evaluated by comparing with results of RT-PCR using NP swabs. Cohen’s weighted kappa index was used to evaluate the agreement between the tests. The non-parametric Mann–Whitney *U*-test was used to compare the difference in the cycle threshold (Ct) values between RT-PCR with NP swabs and RT-PCR with saliva specimens. *P*-values < 0.05 were considered statistically significant. All statistical analyses were performed using Statistical Product and Service Solutions, version 27.0 (IBM, Armonk, NY, USA).

## Results

During the study period, three diagnostic tests were conducted for 157 patients. Of the 157 patients, 124 were about to be hospitalized and 33 were already admitted for COVID-19. Among the 124 patients who were about to be hospitalized, 16 (12.9%) had symptoms compatible with COVID-19, and the most common symptom was sore throat. Among the 33 patients already admitted for COVID-19, 27 (81.8%) had symptoms compatible with COVID-19 at the time of testing, and the most common symptom was cough (n = 13, 39.4%), followed by fever (n = 12, 36.4%).

### Sensitivity and specificity of RAT with nasal swabs compared with RT-PCR with NP swabs

When compared with the RT-PCR results with NP swabs, the overall sensitivity and specificity of the RAT with nasal swabs were 64.7% (95% confidence interval [CI] 47.9–78.5%) and 100.0% (95% CI 97.0–100.0%), respectively (Table 1). The Cohen’s weighted kappa value was 0.74. An analysis of the results using an RT-PCR Ct value ≤ 25 increased the sensitivity to 75.9% (95% CI 57.9–87.8%) (Table 1). In the symptomatic patients (n = 43), the sensitivity of the RAT was 71.4% (95% CI 52.9–84.8%). The median time to confirm a positive RAT result was 180 s (interquartile range 60–255 s; range 60–600 s). We observed test kits over a period of 60 min, and the results did not differ from the initial interpretation.

**Table 1:** The sensitivity and specificity of the Panbio™ COVID-19 Ag rapid test device compared with RT-PCR.

Overall patients (n = 157)				
		RT-PCR		Total
		Positive	Negative	
Panbio™ COVID-19 Ag rapid test device	Positive	22	0	22
	Negative	12	123	135
	Total	34	123	157
Sensitivity = 64.7%; Specificity = 100%; Cohen’s weighted kappa index = 0.74				
Patients with cycle threshold values ≤ 25 (n = 157)				
		RT-PCR		Total
		Positive	Negative	
Panbio™ COVID-19 Ag rapid test device	Positive	22	0	22
	Negative	7	128	135
	Total	29	128	157
Sensitivity = 75.9%; Specificity = 100%; Cohen’s weighted kappa index = 0.84				
Symptomatic patients (n = 43)				
		RT-PCR		Total
		Positive	Negative	
Panbio™ COVID-19 Ag rapid test device	Positive	20	0	20
	Negative	8	15	23
	Total	28	15	43
Sensitivity = 71.4%; Specificity = 100%; Cohen’s weighted kappa index = 0.64				

RT-PCR, reverse transcription-polymerase chain reaction

### Sensitivity and specificity of RT-PCR with saliva compared with RT-PCR with NP swabs

When compared with the RT-PCR results with NP swabs, the overall sensitivity and specificity of the RT-PCR with saliva were 94.1% (95% CI 80.9–98.4%) and 100.0% (95% CI 97.0–100.0%), respectively (Table 2). The Cohen’s weighted kappa value was 0.96. An analysis of the results using an RT-PCR Ct value ≤ 25 increased the sensitivity of the RT-PCR with saliva to 96.6% (95% CI 82.8–99.4%) (Table 2). RT-PCR with NP swabs had a lower Ct value than that of the RT-PCR with saliva specimens (18.5 [15.1–23.9] vs. 23.6 [17.9–29.4]; *P* = 0.015).

**Table 2:** The sensitivity and specificity of SSf-COVID19 kit with saliva compared with RT-PCR with nasopharyngeal swabs

Overall patients (n = 157)				
		Nasopharyngeal swab		Total
		Positive	Negative	
SSf-COVID19 kit	Positive	32	0	32
	Negative	2	123	125
	Total	34	123	157
Sensitivity = 94.1%; Specificity = 100%; Cohen’s weighted kappa index = 0.96				
Patients with cycle threshold values ≤ 25 (n = 157)				
		Nasopharyngeal swab		Total
		Positive	Negative	
SSf-COVID19 kit	Positive	28	4	32
	Negative	1	124	125
	Total	29	128	157
Sensitivity = 96.6%; Specificity = 97.0%; Cohen’s weighted kappa index = 0.90				
Symptomatic patients (n = 43)				
		Nasopharyngeal swab		Total
		Positive	Negative	
SSf-COVID19 kit	Positive	27	0	27
	Negative	1	15	16
	Total	28	15	43
Sensitivity = 96.4%; Specificity = 100%; Cohen’s weighted kappa index = 0.95				

RT-PCR, reverse transcription-polymerase chain reaction

## Discussion

The overall sensitivity of the Panbio™ COVID-19 Ag rapid test device using nasal swab specimens was 64.7% (95% CI 47.9–78.5%). RATs have some benefits in screening, such as rapid identification of test results, low cost, and no requirement of instrument or expert skills [5–7, 11]. However, previous studies have reported that RATs using nasal swabs have lower sensitivity than that of RT-PCR. Peto et al. demonstrated that the sensitivity of RAT with nasal swabs using four different kits, including the Panbio™ COVID-19 Ag rapid test device, ranged from 78.8% as tested by laboratory scientists to 57.5% as tested by self-trained personnel [6]. The results of our study are similar to those of previous studies. The results of RATs for the diagnosis of COVID-19 are available in < 15–30 min. In this study, the results were available within 10 min. Although there have been concerns that interpreting the RAT before or after a specified time could result in false-positive or false-negative test results, this was not observed in our study. Notably, the results of the Panbio™ COVID-19 Ag rapid test device did not differ from the initial interpretation despite the extended observation of 60 min.

Previous reports revealed that saliva specimens could be useful alternatives for detecting SARS-CoV-2 [3, 13, 14]. In a previous study, when compared with the RT-PCR results with NP swabs, the sensitivity and specificity of RT-PCR with saliva specimens were 85.19% and 89.19%, respectively, and the mean Ct value was 28.26 for NP swabs and 32.91 for saliva [13]. Another study revealed that the sensitivity and specificity of RT-PCR with saliva specimens were 94.1% and 98.6%, respectively, and the median Ct values of NP swabs and saliva were 29.4 and 32.7, respectively [14]. In our results, the sensitivity and specificity of RT-PCR using saliva specimens were 94.1% and 100.0%, respectively, and the median Ct values for NP swabs and saliva specimens were 18.5 (15.1–23.9) and 23.6 (17.9–29.4), respectively.

Although RT-PCR using NP swabs is considered the gold standard for diagnosing SARS-CoV-2 infection, it is not useful for repeated screening tests during school re-opening because of its long turn-around time and discomfort in acquiring NP specimens. Repeated rapid and non-invasive tests could be helpful for safe school re-opening. RATs using nasal swabs or RT-PCR using saliva specimens could be considered for repeated screening tests for COVID-19 [11]. In this study, we evaluated the clinical performance of Panbio™ COVID-19 Ag rapid test device and RT-PCR test using self-collected saliva specimens for the detection of SARS-CoV-2 infection.

In conclusion, the sensitivity and specificity of the Panbio™ COVID-19 antigen rapid test device using nasal swabs and those of SSf-COVID19 kit using self-collected saliva specimens were 64.7% and 100% and 94.1% and 100%, respectively.

## Limitations

The small number of patients is a limitation of this study, and further investigations are warranted.

## Data Availability

The data collected and/or analysed during the current study are available from the corresponding author on reasonable request.

## Abbreviations

COVID-19: Coronavirus disease.
NAAT: Nucleic acid amplification tests.
NP: Nasopharyngeal.
RATs: Rapid antigen tests.
RT-PCR: Reverse transcription-polymerase chain reaction.
SARS-CoV-2: Severe acute respiratory syndrome coronavirus 2.
SMG-SNUBMC: Seoul Metropolitan Government-Seoul National University Boramae Medical Center.
SNUH: Seoul National University Hospital.
